# Supplementary material for: Cataract-associated P23T γD-crystallin retains a native-like fold in amorphous-looking aggregates formed at physiological pH
Source: Nat Commun. 2017 May 5;8:15137. doi: 10.1038/ncomms15137 (PMC5424181; doi:10.1038/ncomms15137)
Supplement: Supplementary Information — Supplementary Figures, Supplementary Tables and Supplementary References [file ncomms15137-s1.pdf]

Supplementary Table 1. Detailed experimental conditions of the MAS ssNMR experiments. *Abbreviations: NS, number of scans per t_1 point; Temp., temperature of cooling gas; MAS, magic angle spinning rate; RD, recycle delay; TPPM, ^1H decoupling power during evolution and acquisition using the two-pulse phase modulation scheme* [1].

Fig./ Table	Aggreg. conditions	Expt.	NS	Temp (K)	MAS (kHz)	RD (s)	TPPM (kHz)	t_1 evol. (μs)	t_2 evol. (μs)	Mix. (ms)	H-C/N, C-N CP time (ms)	τ_1, τ_2 (ms)	Exp. time (h)
3a	pH 7	^1H-^{13}C CP	102	298	12.5	3.0	83	NA	NA	NA	2.0, NA	NA	0.8
3b 7a,b S2-3	pH 7	2D ^{13}C-^{13}C CP-DARR	40	283	12.5	2.8	83	34.85* 860	NA	8.0	2.0, NA	NA	54
4a	pH 3	^1H-^{13}C CP	1024	298	12.5	3.0	83	NA	NA	NA	1.5, NA	NA	0.8
4a 7a,b	pH 3	2D ^{13}C-^{13}C CP-DARR	64	298	12.5	2.6	83	37.84* 792	NA	8.0	1.5, NA	NA	70
5a	pH 7	^1H-^{13}C INEPT	256	298	8.333	2.8	83	NA	NA	NA	NA, NA	1.5, 1.0	0.2
5b	pH 3	2D ^{13}C-^{13}C TOBSY	64	298	8.333	2.8	83	50.94* 400	NA	6.0	NA, NA	1.2, 1.0	20
S3	pH 7	3D NCACX	80	298	12.5	2.5	83	410.76 * 16	275.89 *24	15	2.0, 6.0	NA	87.0
S3	pH 7	3D NCOCX	80	298	12.5	2.5	83	410.76 * 16	275.89 *24	30	2.5, 5.5	NA	87.5
Table S2	pH 7	2D NCACX	112	298	10.0	3.0	83	100.00 * 100	NA	25	1.5, 6.0	NA	19.0

Supplementary Table 2: Chemical shifts of residues assigned in amorphous P23T hγD aggregates at physiological pH determined by MAS ssNMR. *The reported ^{15}N and ^{13}C chemical shifts are referenced relative to liquid ammonia and dilute aqueous DSS, respectively (see methods), and have an experimental error of 0.4 ppm for ^{13}C and 0.5 ppm for ^{15}N.*

Res. Num.	Type	N	C'	Cα	Cβ	Cγ1	Cγ2	Cδ	Cε
1	Gly		169.5	43.6					
2	Lys	124.4	172.9	57.8	35.7	24.6		26.6	42.1
3	Ile	128.6	171.5	58.9	41.3	28.5	15.3	12.9	
4	Thr	122.5	172.5	61.8	69.2	20.9			
5	Leu	128.2	175.1	53.7	44.1	27.4			
23[a]	Thr	113.2	176.4	64.3	72.3	22.9			
26	Gln	119.5	174.0	59.5	28.2				
27	Pro	130.6	175.7	64.6	30.4	28.0		50.7	
34	Ser	107.9	173.6	58.8	64.4				
35	Ala	118.9	175.4	52.7	22.8				
36	Arg	123.8	175.5	54.9	33.9				
37	Val	131.8	175.5	62.2	31.0	23.2			
62[b]	Tyr	122.6	174.4	56.3*	39.8*			132.2	
63	Ala	126.5	175.7	53.8	19.3				
80	Leu	126.0	175.7	53.6	41.8	26.8			
81	Ile	130.3	173.8	60.0	42.2	27.7	18.6		
82	Pro	138.1	177.1	63.6	32.3	26.4		51.3	
89	Ile	130.0	170.7	59.1	42.1	28.1	15.7*	14.9	
98	Arg	117.7	175.2	54.4	33.1	27.5			
99	Gly	105.2	172.5	44.0					
100[b]	Gln	119.6	174.2	57.7*	29.8*	32.6			
101	Met	120.0	175.3	53.4	36.1				
102	Ile	124.0	171.0	60.4	41.4	28.2	15.5	12.9	
103	Glu	127.4	175.3	53.7	33.8	37.5		183.6	
104	Phe	120.8	176.1	57.6	43.5				
105[b]	Thr	108.8	173.3	61.0*	70.5*	21.5			
107	Asp	115.8	176.4	55.5	41.4				
108	Cys	121.3	174.2	57.8	28.7				
109[b]	Ser	124.5	173.9	60.9*	62.8*				
120[c]	Ile	122.7	173.9	57.9	38.7	26.9*	19.4	11.5*	
156	Trp	114.9	176.6	57.1	28.2				
157	Gly	108.0	173.4	46.1					
158	Ala	120.0	177.0	51.4	22.2				
159	Thr	106.3	175.4	61.3	70.7	22.2			

Res. Num.	Type	N	C'	Cα	Cβ	Cγ1	Cγ2	Cδ	Cε
160	Asn	117.2	171.7	52.1	39.5	176.5			
161	Ala	119.6	176.4	51.4	19.3				
162	Arg	119.8	176.9	58.6	29.6				
163	Val	120.7	174.4	61.7	36.4				
169	Val	124.3	174.7	65.2	30.0	21.8	20.7		
170	Ile	130.1	173.9	58.1	40.4	25.3	20.2	17.0	

[a] Residue T23 was assigned to these chemical shifts on the basis that the other three (out of four) Thr residues were unambiguously assigned in the 2D and 3D ssNMR spectra.
[b] Atoms labeled with asterisk (*) are tentatively assigned based on close correspondence between the solution NMR and solid state NMR shifts[2].
[c] Resonances for residue I120 were assigned by exclusion since the other five (out of six) Ile residues were unambiguously assigned from the 2D and 3D ssNMR spectra. This tentative assignment is further supported by the characteristic pattern that sets I120 apart from the other Ile residues in the solution NMR data.

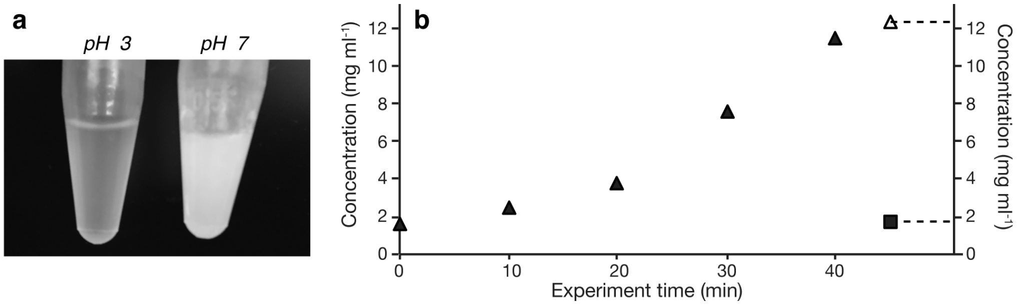

Supplementary Figure 1: P23T hγD aggregation. (a) P23T hγD aggregates. Left: 1.3 mg ml^{-1} suspension of aggregates formed at pH 3. Right: 9.2 mg ml^{-1} suspension of aggregates formed at pH 7. (b) Concentration-dependent aggregation of P23T hγD at neutral pH. Starting with an initial concentration of 1.8 mg ml^{-1}, the solution was concentrated at 4 °C using a centrifugal concentrator. Triangles mark the total protein concentration determined by UV-VIS spectrometry at different times (solid) with the open triangle representing the concentration at the onset of aggregation (>12 mg ml^{-1}). The residual monomer concentration after completion of aggregation and removal of aggregates (1.7 mg ml^{-1}) is shown by the solid square. Note that the solubility of wild-type hγD is at least one to two orders of magnitude larger, with reported solubilities ranging from 170 to more than 400 mg ml^{-1}, depending on conditions[3, 4].

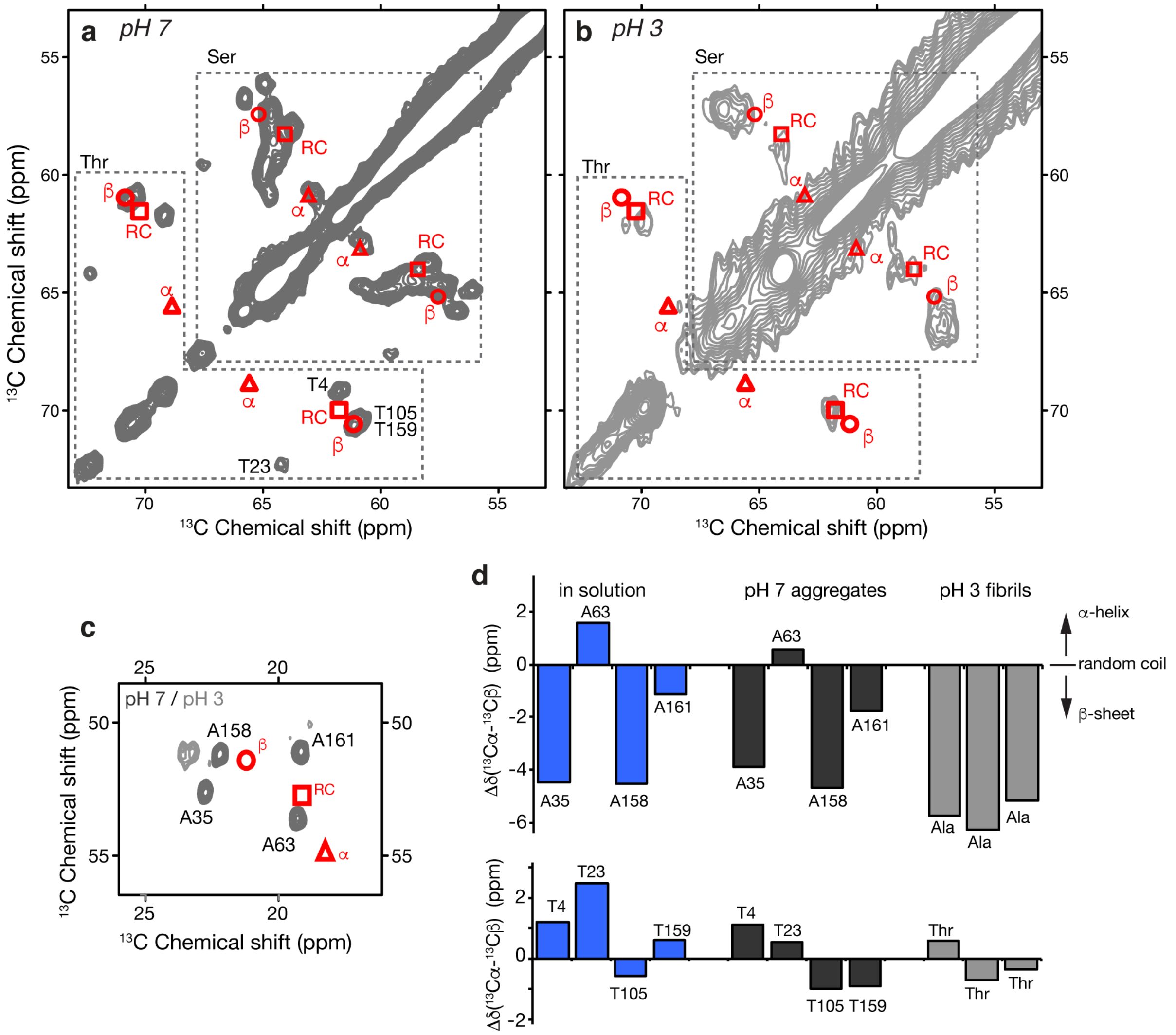

Supplementary Figure 2: NMR chemical shift analysis of residue-specific secondary structure. (a) Thr and Ser Cα/Cβ region of the 2D ^{13}C-^{13}C ssNMR spectrum of P23T hγD aggregated at neutral pH. The expected peak positions[5] for Thr and Ser in random coil (RC), α-helix (α) and β-sheet (β) structural motifs are indicated by red symbols superimposed on the experimental spectrum (grey). Peak assignments are based on 2D and 3D ssNMR spectra (see Supplementary Table 2). (b) Analogous representation for the acid-induced P23T hγD fibrils. (c) Superposition of the Ala Cα/Cβ cross-peak region of the 2D ^{13}C-^{13}C spectra of aggregates formed at pH 7 (dark grey) and pH 3 (light grey), along with the expected peak positions for Ala in different secondary structures. Indicated peak assignments are based on 2D and 3D ssNMR spectra (see Supplementary Table 2). (d) Secondary chemical shifts ($\Delta\delta(^{13}C\alpha\text{-}^{13}C\beta)$) calculated for the Ala (top) and Thr (bottom) residues of P23T hγD in solution (left)[2], aggregated at pH 7 (middle), or at pH 3 (right). These secondary shifts, $\Delta\delta(^{13}C\alpha\text{-}^{13}C\beta)$, are normalized relative to the shift of a random coil conformation, such that negative values are indicative of β-sheet structure, whereas positive bars indicate α-helical structure (as indicated on far right).

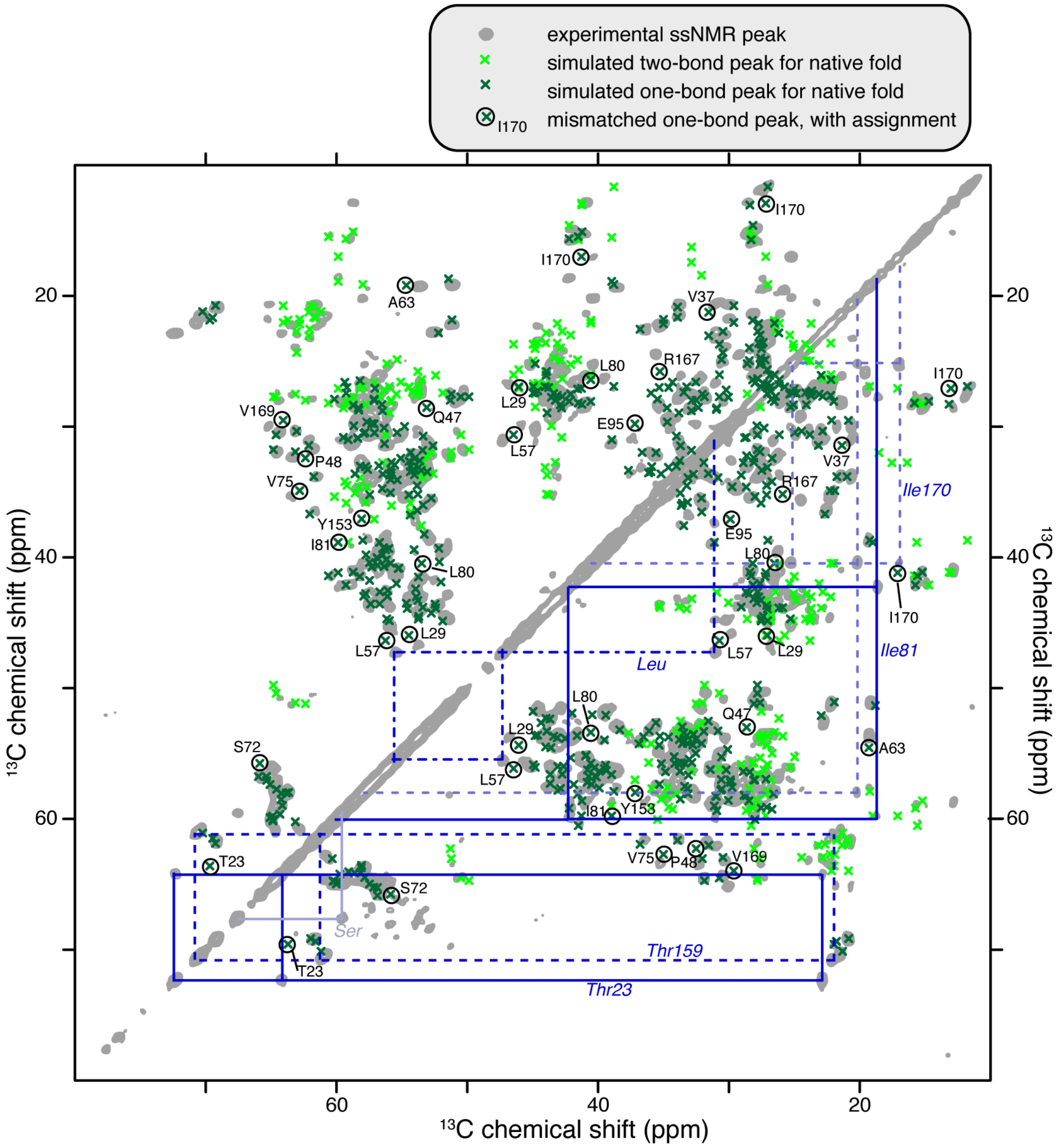

Supplementary Figure 3: Experimental ^{13}C-^{13}C ssNMR spectrum of amorphous P23T hγD aggregates annotated with the liquid-state resonance frequencies of P23T hγD in solution. The grey spectrum is a 2D ^{13}C-^{13}C MAS ssNMR spectrum with 8 ms of ^{13}C-^{13}C DARR mixing. The off-diagonal peaks arise mostly from one-bond ^{13}C-^{13}C correlations, along with a few two-bond contacts. Dark and light green x symbols indicate one- and two-bond correlations, respectively, predicted from the solution NMR assignments of soluble P23T hγD[2]. Well-resolved one-bond solution correlations that do not coincide with cross-peaks in the ssNMR spectrum are labeled with ⊗ symbols, as well as the (singe-letter) residue names and numbers. Conversely, correlated sets of ssNMR peaks that do *not* overlap with any corresponding solution NMR frequencies are connected by dashed or continuous blue lines and labeled with residue names and numbers (three letter codes; see ssNMR assignments in Supplementary Table 2 and Supplementary Fig. 4).

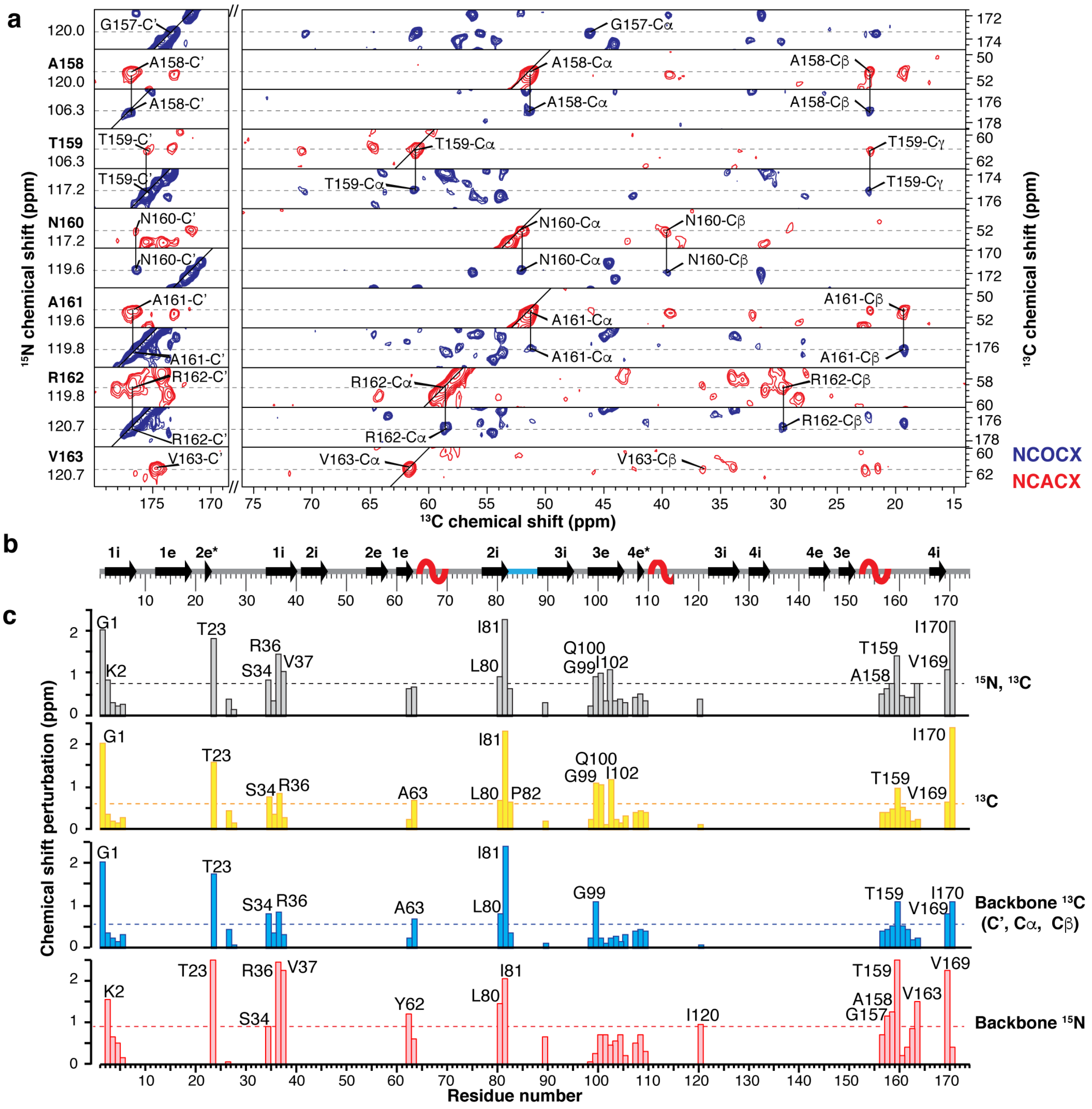

Supplementary Figure 4: MAS ssNMR chemical shift assignments of amorphous P23T hγD aggregates formed at physiological pH. (a) Sequential backbone walk for residues A158-V163 based on 3D NCOCX (navy) and 3D NCACX (red) spectra. (b) Secondary structure content of the solution-state structure [2]. The β-strands (arrows) and single-residue β-bridges (*) are labeled as internal (i) or external (e) within Greek key motifs 1-4, while α-helices are marked as red helices. The linker between the NTD and CTD is shown in blue. (c) Chemical shift perturbation (CSP) of P23T hγD upon formation of amorphous aggregates, based on the difference between the assigned ssNMR shifts (Supplementary Table 2) and the P23T hγD solution NMR chemical shifts [2]. Bars indicate the combined RMSD of ^{13}C and ^{15}N atoms, ^{13}C, backbone ^{13}C (C', Cα and Cβ) and backbone ^{15}N, respectively. The average RMSDs of 0.75, 0.55, 0.62, and 0.91 ppm are plotted as dashed lines. No ^{15}N solution NMR shifts were available for G1, P27, and P82.

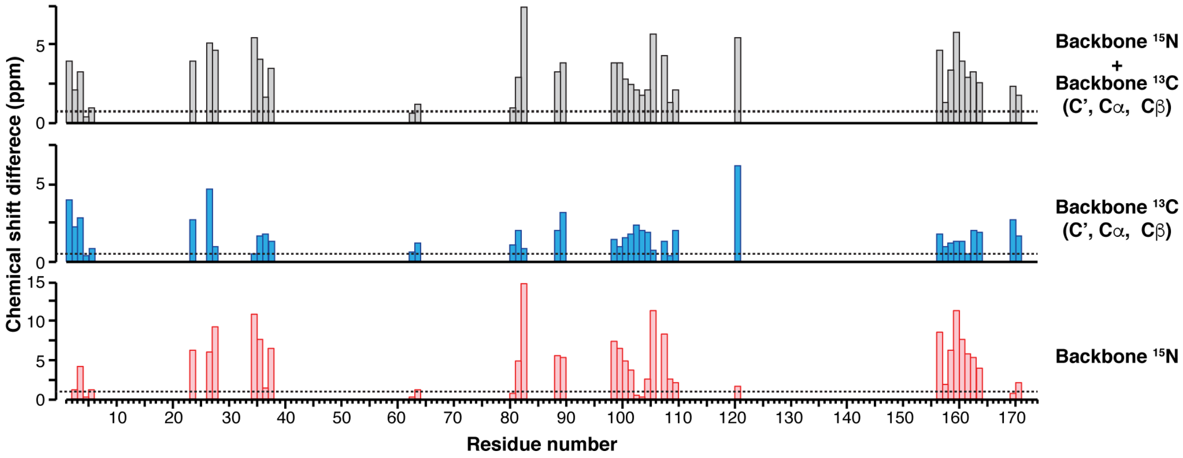

Supplementary Figure 5: MAS ssNMR chemical shift differences between amorphous P23T hγD aggregates and the simulated amyloid-like model. Bars show the chemical shift differences between the experimental chemical shifts of the amorphous P23T hγD aggregates (Supplementary Table 2) and the chemical shifts predicted for our schematic amyloid-like model (Fig. 6a). Shown are the combined RMSD of the backbone ^{13}C and ^{15}N atoms, the backbone ^{13}C (C', Cα and Cβ) and backbone ^{15}N, respectively. The employed prediction algorithm does not produce the ^{13}C shifts of the entire side chain, leading us to omit the full-residue ^{13}C chemical shift deviations present in Supplementary Fig. 4. Note the different scale of the vertical axes compared to Supplementary Fig. 4, reflecting the much larger chemical shift deviation between the schematic amyloid-like model and the observed ssNMR spectra. For comparison, the average RMSDs from Supplementary Fig. 4 are included as dashed lines.

References cited in the Supporting Information.

1. Bennett, A. E., Rienstra, C. M., Auger, M., Lakshmi, K. V. & Griffin, R. G. Heteronuclear decoupling in rotating solids. *J. Chem. Phys.* **103**, 6951-6958 (1995).

2. Jung, J., Byeon, I.-J. L., Wang, Y., King, J. & Gronenborn, A. M. The structure of the cataract-causing P23T mutant of human gammaD-crystallin exhibits distinctive local conformational and dynamic changes. *Biochemistry* **48**, 2597-2609 (2009).

3. Evans, P., Wyatt, K., Wistow, G. J., Bateman, O., Wallace, B. A. & Slingsby, C. The P23T cataract mutation causes loss of solubility of folded gammaD-crystallin. *J. Mol. Biol.* **343**, 435-444 (2004).

4. Pande, A., Annunziata, O., Asherie, N., Ogun, O., Benedek, G. B. & Pande, J. Decrease in protein solubility and cataract formation caused by the Pro23 to Thr mutation in human gamma D-crystallin. *Biochemistry* **44**, 2491-2500 (2005).

5. Zhang, H., Neal, S. & Wishart, D. S. RefDB: a database of uniformly referenced protein chemical shifts. *J. Biomol. NMR* **25**, 173-195 (2003).
